# Kinesin family member 18B regulates the proliferation and invasion of human prostate cancer cells

**DOI:** 10.1038/s41419-021-03582-2

**Published:** 2021-03-22

**Authors:** Yu-Peng Wu, Zhi-Bin Ke, Wen-Cai Zheng, Ye-Hui Chen, Jun-Ming Zhu, Fei Lin, Xiao-Dong Li, Shao-Hao Chen, Hai Cai, Qing-Shui Zheng, Yong Wei, Xue-Yi Xue, Ning Xu

**Affiliations:** Department of Urology, Urology Research Institute, the First Affiliated Hospital, Fujian Medical University, Fuzhou, 350005 China

**Keywords:** Prostate cancer, Cell invasion

## Abstract

Expression of kinesin family member 18B (KIF18B), an ATPase with key roles in cell division, is deregulated in many cancers, but its involvement in prostate cancer (PCa) is unclear. Here, we investigated the expression and function of KIF18B in human PCa specimens and cell lines using bioinformatics analyses, immunohistochemical and immunofluorescence microscopy, and RT-qPCR and western blot analyses. KIF18B was overexpressed in PCa specimens compared with paracancerous tissues and was associated with poorer disease-free survival. In vitro, KIF18B knockdown in PCa cell lines promoted cell proliferation, migration, and invasion, and inhibited cell apoptosis, while KIF18B overexpression had the opposite effects. In a mouse xenograft model, KIF18B overexpression accelerated and promoted the growth of PCa tumors. Bioinformatics analysis of control and KIF18B-overexpressing PCa cells showed that genes involved in the PI3K–AKT–mTOR signaling pathway were significantly enriched among the differentially expressed genes. Consistent with this observation, we found that KIF18B overexpression activates the PI3K–AKT–mTOR signaling pathway in PCa cells both in vitro and in vivo. Collectively, our results suggest that KIF18B plays a crucial role in PCa via activation of the PI3K–AKT–mTOR signaling pathway, and raise the possibility that KIF18B could have utility as a novel biomarker for PCa.

## Introduction

Prostate cancer (PCa) is one of the most common non-cutaneous malignancies among men in the United States, and is expected to be responsible for an estimated 33,330 deaths in 2020^1^. Metastasis is the predominant cause of mortality in PCa, largely because it is often present at the time of diagnosis and can develop rapidly after failure of initial treatment, which includes surgery and/or radiotherapy^2^. Consequently, it is crucial that we identify novel markers that can accurately demarcate or predict PCa disease stage and prognosis, as well as novel targets to effectively control the disease and improve prognosis^3^.

The kinesins are a > 40-member superfamily of genes that encode ATPases involved in the regulation of microtubule-associated motor proteins. As such, kinesins are known to play crucial roles in numerous key cellular processes, including meiosis, mitosis, and autophagy. Overexpression of kinesins as been shown to be closely associated with the development and growth of human tumors, and KIF18B upregulation is therefore likely to affect patient survival^4–6^. For example, De et al. suggested that overexpression of the kinesins KIFC1, KIF1A, KIF5A, and KIFC3 increased the resistance of breast cancer cells to docetaxel chemotherapy^7^. Ishikawa et al. reported that KIF2C was overexpressed in 120 patients with colorectal cancer and identified the KIF2C gene as a novel marker of poor prognosis and lymph node metastasis in these patients^8^. In addition, a lack of KIF4 expression has been associated with increased aneuploidy in mouse embryonic stem cells as well as tumor formation in nude mice^9^.

The kinesin family member KIF18B has been implicated in the separation and pairing of chromosomes during mitosis^10^. Although an association between KIF18B and cancer has been reported^11–13^, the role of this protein in tumorigenesis remains poorly understood. A bioinformatics analysis by Itzel et al. first demonstrates that KIF18B overexpression correlates with poor survival outcomes in patients with hepatic carcinoma^14^. Yaqin et al. also identified KIF18B as a potential disease biomarker and oncogene in cervical cancer, and demonstrated roles for KIF18B in promoting cell invasion and proliferation in vitro and in vivo^15^.

The progression of PCa is a complex process involving numerous molecular pathways, of which the phosphatidylinositol 3-kinase–AKT–mammalian target of rapamycin (PI3K–AKT–mTOR) signaling pathway is the key regulatory pathway^16,17^. Lamoureux et al.^18^ demonstrated that PI3K–AKT pathway activation is associated with poor clinical outcomes of PCa patients; similarly, Kreisberg et al.^19^ showed that activation (phosphorylation) of AKT is a predictor of poor clinical outcome in PCa. Nevertheless, the role(s) of KIF18B in PCa remain relatively unexplored.

In the present study, we sought to address the urgent for novel molecular biomarkers in PCa that will facilitate disease management, accurately predict clinical outcomes, and serve as effective targets. We examined correlations between KIF18B expression in patient specimens and various clinicopathological factors, and additionally explored the potential function of KIF18B using cultured human PCa cell lines. Our results demonstrate that KIF18B is overexpressed in PCa, promotes PCa cell invasion and proliferation, and may have clinical utility as a potential biomarker for this disease.

## Results

### WGCNA and co-expression analyses

**Fig. 1:**
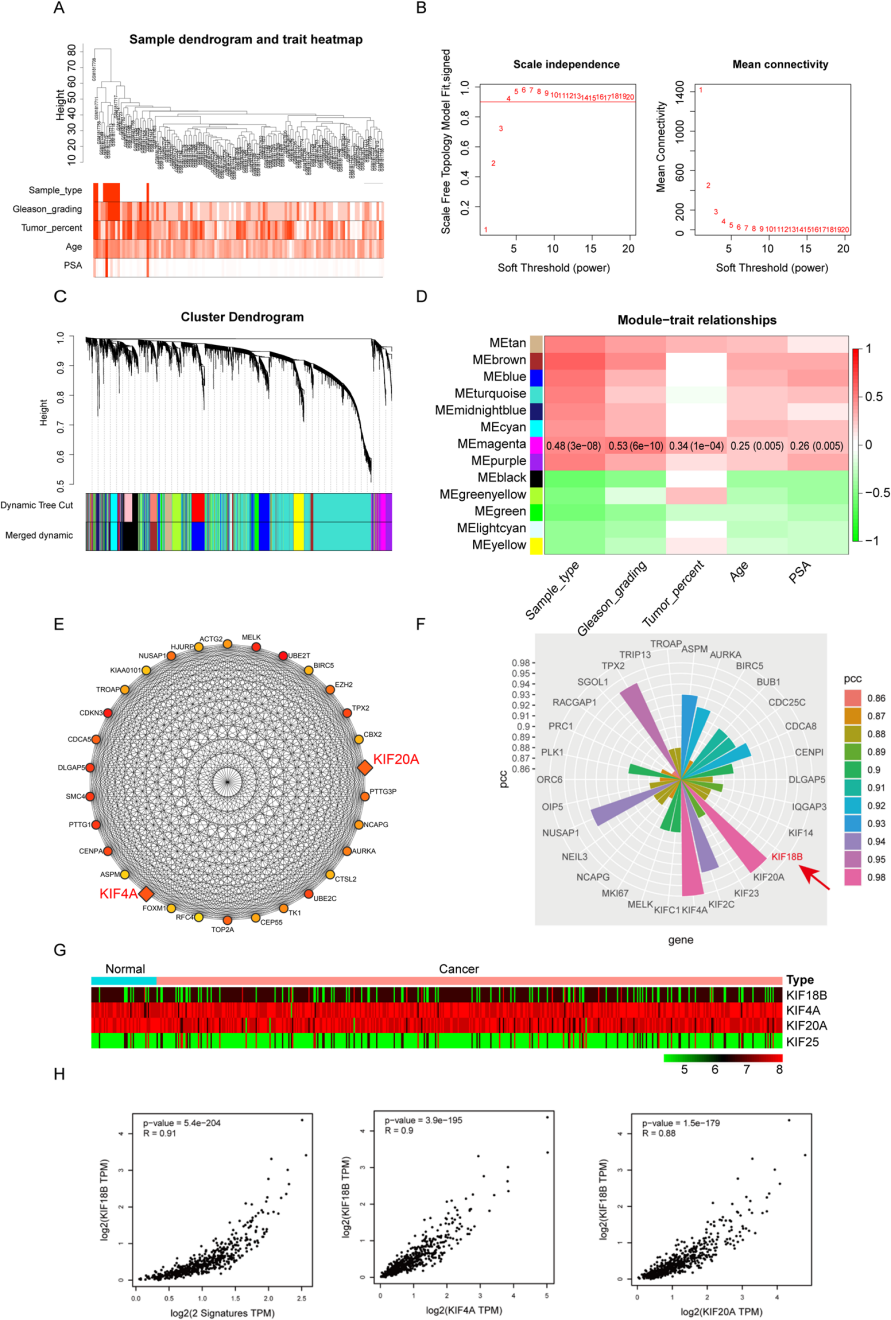
Bioinformatics analysis demonstrates a key role for KIF18B in the development and progression of PCa. **A** Sample dendrogram shows the samples included in this study and trait heatmap shows the distribution of the clinicopathological characteristics among these samples. **B** Scale independence and mean connectivity demonstrates the best soft threshold was determined as 4. **C** Cluster dendrogram shows the processes of screening of modules. Each of the modules were determined by dynamic tree cut method first and then merged finally. **D** Module-trait relationships demonstrate the relationships between the clinicopathological characteristics and selected modules. Pearson coefficient and *P* value were utilized for screening the interested modules. **E** The PPI network of genes in magenta modules. KIF20A and KIF4A were the dominant genes in the PPI network. **F** Nightingale’s Rose diagram demonstrated the co-expression genes with KIF18B. **G** The heatmaps demonstrated differentially expressed KIF-related genes in PCa. **H** The expression correlation analyses demonstrated the expression pattern of KIF18B with KIF20A and KIF4A. Spearman coefficient and *P* value were utilized for evaluating the expression correlation.

A sample dendrogram and trait heatmap are shown in Fig. 1A. A total of 13 modules were selected with a soft threshold of 4 (Fig. 1B, C); of these, the magenta module had the highest correlation with clinicopathological characteristics (Fig. 1D). The PPI network demonstrated that members of the kinesin family were key genes in the magenta module (Fig. 1E). A TCGA PCa dataset was used to validate the expression of kinesin family genes in PCa, and the analysis showed that KIF18B, KIF4A, KIF20A, and KIF25 were significantly differentially expressed in PCa compared with normal prostate tissue (Fig. 1F). This is consistent with previous demonstrations that KIF4A and KIF20A are significantly associated with the progression of PCa. Co-expression and correlation analyses revealed that the expression pattern of KIF18B was similar to and significantly correlated with the expression patterns of KIF4A and KIF20A (Fig. 1G, H).

### KIF18B expression is elevated in human PCa tissues

**Fig. 2:**
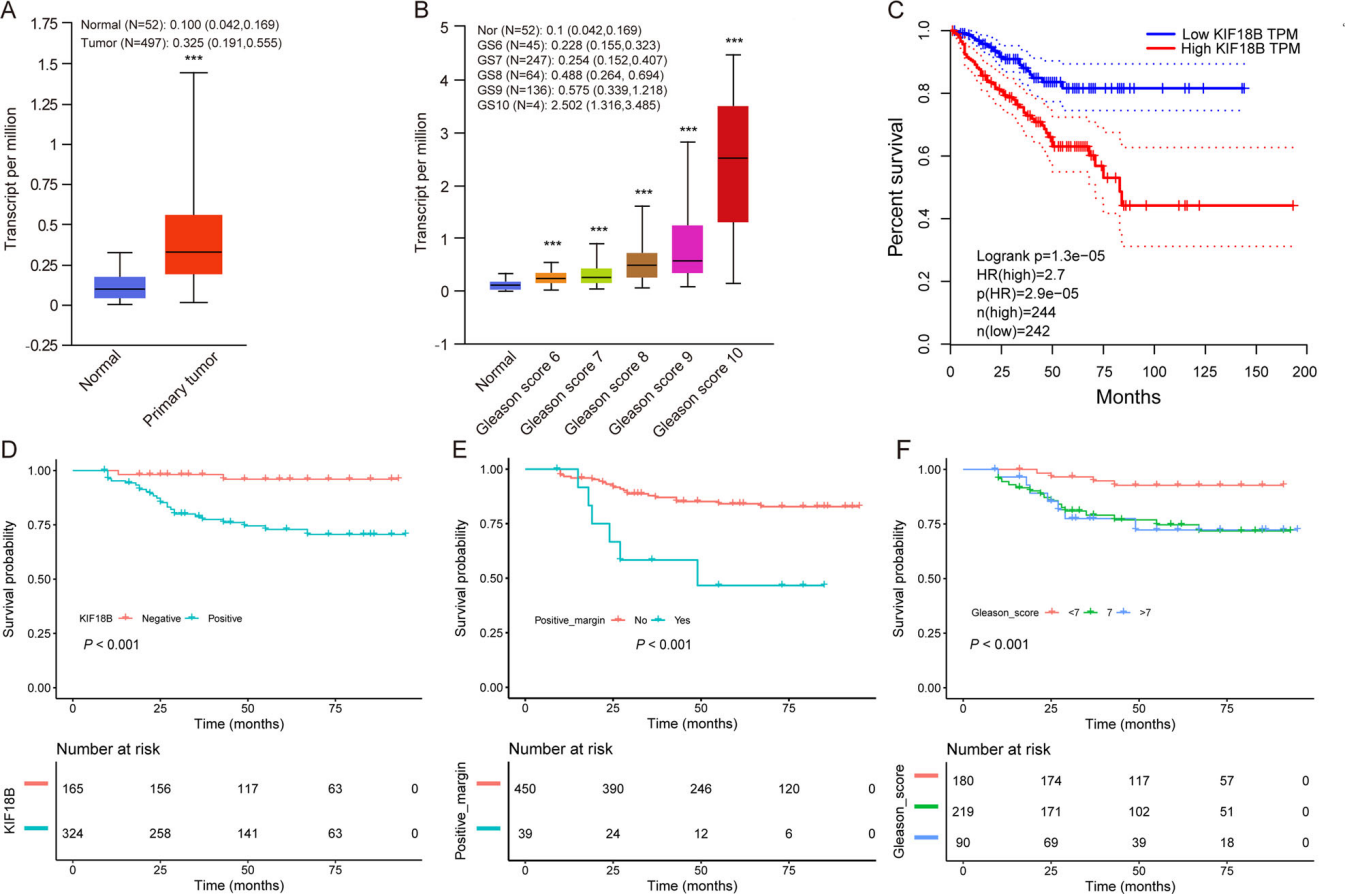
Integrated analysis of the relationship between KIF18B expression and clinicopathological characteristics in PCa. **A** KIF18B expression in PCa and normal patient tissue samples from the TCGA dataset (normal, *n* = 52; tumor, *n* = 497). **B** KIF18B expression in PCa according to the Gleason score (normal, *n* = 52; score 6, *n* = 45; score 7, *n* = 247; score 8, *n* = 64; score 9, *n* = 136; score 10, *n* = 4). *P* < 0.001 for the following comparisons: normal vs Gleason score 7, normal vs score 8, normal vs score 9, score 6 vs score 7, score 6 vs score 8, score 6 vs score 9, score 7 vs score 8, and score 7 vs score 9. **C** Cancer-free survival analysis of patients in the TCGA dataset according to KIF18B mRNA expression level (high, *n* = 244; low, *n* = 242). **D**–**F** Biochemical recurrence-free survival analysis of patients stratified by **D** KIF18B expression (negative, *n* = 165; positive, *n* = 324), **E** positive surgical margin status (no, *n* = 450; yes, *n* = 39), and **F** Gleason score (<7, *n* = 180; 7, *n* = 219; >7, *n* = 90). Statistical analyses were performed by UALCAN (**A**, **B**) or the log-rank test (**D**–**F**). Boxplot data are presented as the median (Q1, Q3). ^***^P < 0.001.

Analysis of the TCGA dataset indicated that KIF18B mRNA levels are significantly elevated in PCa tissues compared with non-tumor adjacent tissues (Fig. 2A). Notably, KIF18B was significantly elevated in tumors from patients with Gleason scores 7, 8, and 9 compared with normal tissues and compared with tumors with Gleason score 6. Thus, significant differences (all *P* < 0.001) in KIF18B levels were observed for normal tissue vs Gleason score 7, 8, and 9; score 6 vs score 7, score 6 vs score 8, score 6 vs score 9; score 7 vs score 8, and score 7 vs score 9 (Fig. 2B). Moreover, Kaplan–Meier survival analyses demonstrated that high KIF18B expression was significantly associated with poorer prostate cancer-free survival (Fig. 2C). Comparison of KIF18B expression levels based on the PTEN gene mutation status demonstrated that KIF18B was significantly higher in PCa tumors from patients harboring mutant PTEN compared with those harboring wild-type PTEN (Supplementary Fig. 1). Analysis of the association between KIF18B expression and patient clinicopathological characteristics revealed significant associations with biochemical recurrence, prostate volume, prostate-specific antigen density (PSAD), Gleason score, and positive margin (all *P* < 0.05; Table 1).

**Table 1:** Clinicopathological characteristics of patients stratified by KIF18B expression.

Variables	Negative	Positive	*P*-value
*N*	165	324	
Biochemical recurrence			<0.001
No	159 (96.364%)	246 (75.926%)	
Yes	6 (3.636%)	78 (24.074%)	
Age (years)			0.334
<70	84 (50.909%)	150 (46.296%)	
≥70	81 (49.091%)	174 (53.704%)	
Prostate volume (cm^3^)			<0.001
≤35	78 (47.273%)	99 (30.556%)	
>35	87 (52.727%)	225 (69.444%)	
PSA (ng/ml)			0.832
<10	30 (18.182%)	57 (17.593%)	
10–20	111 (67.273%)	213 (65.741%)	
>20	24 (14.545%)	54 (16.667%)	
PSAD (ng/ml cm^3^)			0.002
<0.15	3 (1.818%)	30 (9.259%)	
≥0.15	162 (98.182%)	294 (90.741%)	
Gleason score			<0.001
<7	96 (58.182%)	84 (25.926%)	
7	51 (30.909%)	168 (51.852%)	
>7	18 (10.909%)	72 (22.222%)	
Positive margin			0.011
No	159 (96.364%)	291 (89.815%)	
Yes	6 (3.636%)	33 (10.185%)	

Data were analyzed using chi-square test.

PSA prostate-specific antigen, PSAD prostate-specific antigen density.

### KIF18B expression is associated with biochemical recurrence-free survival of PCa patients

We performed univariate and multivariate Cox proportional hazards analyses to determine risk factors for predicting biochemical recurrence-free survival. In multivariate analysis, Gleason ≥7, positive surgical margin, and positive KIF18B expression were significant risk factors for poor biochemical recurrence-free survival (*P* < 0.05, Table 2). Kaplan–Meier analyses confirmed the associations between these variables (Fig. 2D–F, *P* < 0.05).

**Table 2:** Univariate and multivariate Cox regression analyses of risk factors for biochemical recurrence.

Variable	Univariate (coef, HR (95% CI), *P* value)	Multiple Cox regression (coef, HR (95% CI), *P* value)
Age (years)		
<70	1	1
≥70	0.468, 1.593 (1.022, 2.496), 0.040	0.3751, 1.455 (0.855, 2.392), 0.1389
Prostate volume (cm^3^)		
≤35	1	
>35	0.405, 1.500 (0.934, 2.409), 0.0934	
PSA (ng/ml)		
<10	1	
10–20	0.700, 2.013 (1.001, 4.049), 0.0500	
>20	0.425, 1.529 (0.644, 3.630), 0.336	
PSAD (ng/ml cm^3^)		
<0.15	1	
≥0.15	0.623, 1.864 (0.588, 5.905), 0.290	
Gleason score		
<7	1	1
7	1.464, 4.324 (2.304, 8.116), <0.001	1.021, 2.777 (1.469, 5.247), 0.0017
>7	1.500, 4.461 (2.194, 9.074), <0.001	0.857, 2.355 (1.027, 5.401), 0.0430
pT stage		
pT2	1	
pT3a	0.665, 1.944 (0.956, 3.950), 0.0663	
pT3b	1.884, 6.576 (3.052, 14.171), <0.001	
Positive margin		
No	1	1
Yes	1.440, 4.222, (2.503, 7.122), <0.001	1.015, 2.758 (1.474, 5.167), 0.0015
KIF18B		
Negative	1	1
Positive	2.1322, 8.433 (3.671, 19.371), <0.001	1.882, 6.569 (2.829, 15.255), <0.001

Values are expressed as the correlation coefficient (coef), hazard ratio (HR), and 95% confidence intervals (CI).

PSA prostate-specific antigen, PSAD prostate-specific antigen density.

### Validation of KIF18B expression in PCa and paracancerous tissues

**Fig. 3:**
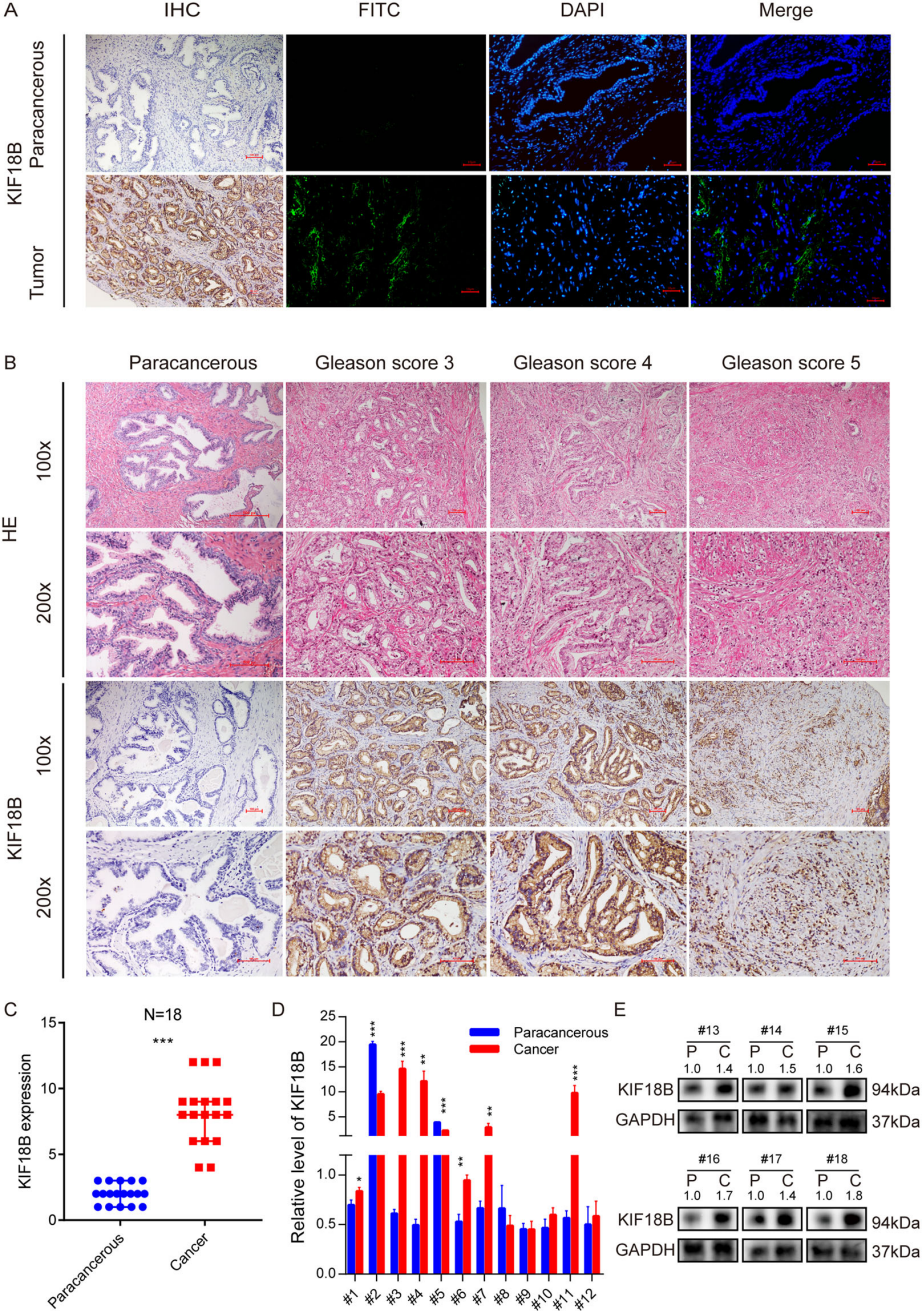
KIF18B expression is upregulated in PCa tissues. **A** Representative immunohistochemical and immunofluorescence images of KIF18B expression in paracancerous and PCa tissues. Scale bar, 10 µm. **B** Representative images of KIF18B expression in paracancerous and PCa tissues from patients with Gleason scores 3, 4, and 5. Scale bar, 100 µm. **C** Quantitative analysis of relative KIF18B expression level in paracancerous and PCa tissues (*n* = 18). **D** RT-qPCR analysis of KIF18B mRNA levels in paracancerous and PCa tissues (*n* = 12). **E** Western blot analysis of KIF1B protein levels in paracancerous and PCa tissues (*n* = 6). Boxplot data are presented as the median (Q1, Q3) and were analyzed with the Mann–Whitney U test. Bar chart data are presented as the mean ± SD of at least three independent experiments and were analyzed with Student’s *t* test (^*^P < 0.05, ^**^P < 0.01, ^***^P < 0.001).

To validate the results obtained with the TCGA dataset, we performed IHC and IF microscopy to assess KIF18B protein expression in matched PCa and adjacent paracancerous tissues. These analyses demonstrated significant higher expression of KIF18B in PCa tissues compared with paracancerous tissues (Fig. 3A). Moreover, KIF18B was overexpressed in tissues from patients with Gleason scores 3–5 compared with paracancerous tissues (Fig. 3B, C). Furthermore, RT-qPCR and Western blot analyses confirmed the upregulation of KIF18B mRNA (*n* = 12 pairs, Fig. 3D) and KIF18B protein (*n* = 6 pairs, Fig. 3E) in PCa tissues compared with matched paracancerous tissues.

### KIF18B promotes the proliferation of DU145 and PC-3 cells

**Fig. 4:**
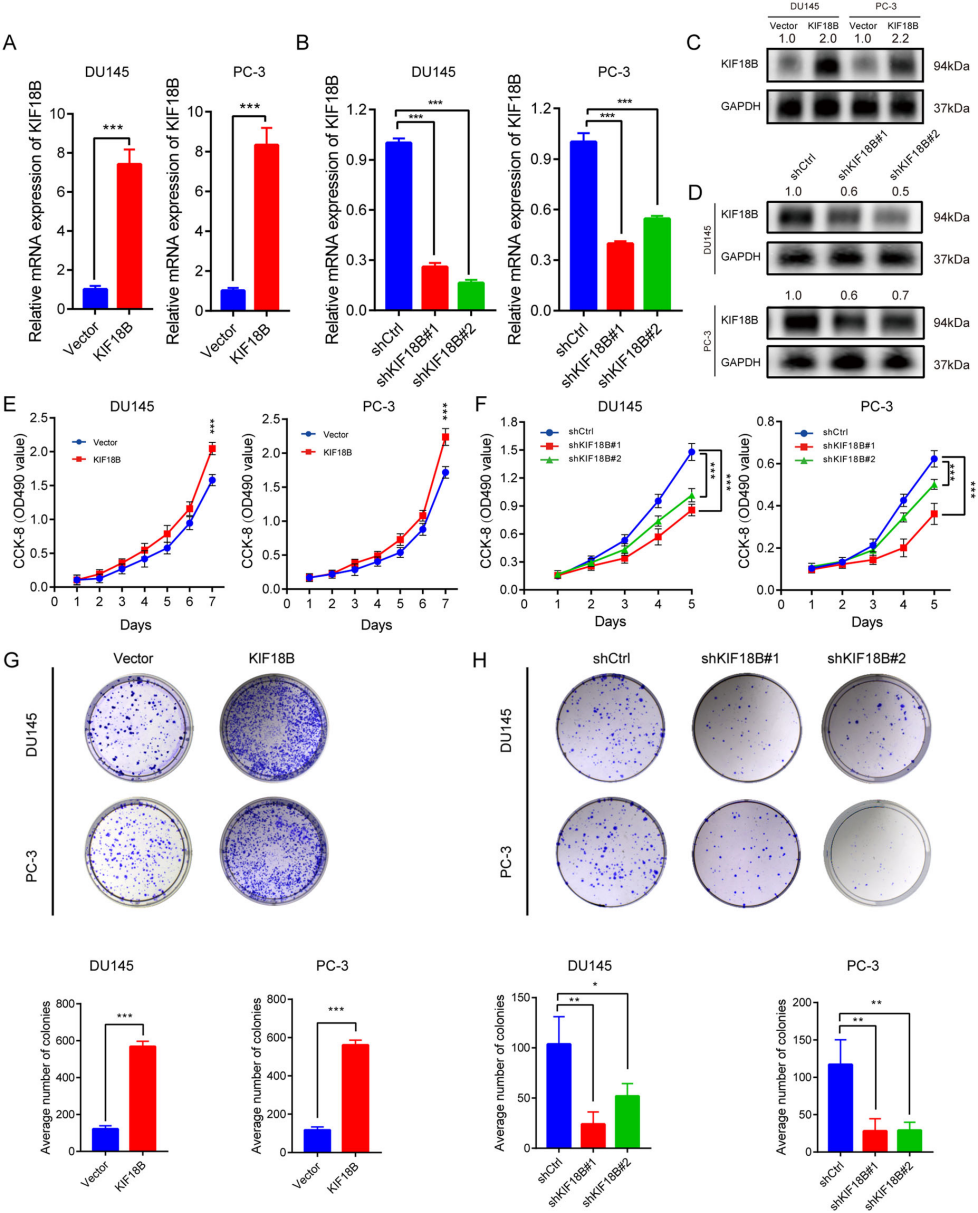
KIF18B promotes DU145 and PC-3 cell proliferation. **A**, **B** RT-qPCR analysis of KIF18B mRNA levels in **A** control and stable KIF18B-overexpressing DU145 and PC-3 cells and **B** DU145 and PC-3 cells transfected with shCtrl, shKIF18B#1, or shKIF18B#2. **C**, **D** Western blot analysis of KIF18B protein levels in **C** KIF18B-overexpressing DU145 and PC-3 cells and **D** DU145 and PC-3 cells transfected with shCtrl, shKIF18B#1, or shKIF18B#2. **E**, **F** CCK-8 cell viability assays of **E** KIF18B-overexpressing DU145 and PC-3 cells and **F** DU145 and PC-3 cells transfected with shCtrl, shKIF18B#1, or shKIF18B#2. **G**, **H** Colony formation assays of **G** KIF18B-overexpressing DU145 and PC-3 cells and **H** DU145 and PC-3 cells transfected with shCtrl, shKIF18B#1, or shKIF18B#2. Data are presented as the mean ± SD of at least three independent experiments and were analyzed with Student’s *t* test or one-way ANOVA (^*^P < 0.05, ^**^P < 0.01, ^***^P < 0.001).

To further investigate the function of KIF18B in PCa, we performed in vitro analyses with the human PCa cell lines DU145, PC-3, and LNCaP (Supplementary Fig. 2). The highest expression of KIF18B detected by RT-qPCR analysis was observed in PC-3 cells, followed by LNCaP and then DU145 cells. Therefore, we selected DU145 and PC-3 cell lines for experiments in which KIF18B is overexpressed or silenced. Stable cell lines were established by lentivirus-mediated transduction with control or KIF18B overexpression plasmids and control (shCtrl) or KIF18B-targeting short hairpin RNAs (shKIF18B#1 and shKIF18B#2). RT-qPCR analysis of the stable cell lines revealed relative KIF18B mRNA expression levels of 1.00 ± 0.17 and 7.42 ± 0.76 for control and KIF18B-overexpressing DU145 cells, respectively (*P* < 0.001) and 1.00 ± 0.14 and 8.33 ± 0.87 for control and KIF18B-overexpressing PC-3 cells, respectively (*P* < 0.001) (Fig. 4A). Conversely, mRNA levels in shCtrl-, shKIF18B#1-, and shKIF18B#2- expressing were 1.00 ± 0.028, 0.26 ± 0.24, and 0.16 ± 0.019, respectively (*P* < 0.001) for DU145 cells and 1.00 ± 0.052, 0.398 ± 0.015, and 0.55 ± 0.017, respectively (*P* < 0.001) for PC-3 cells (Fig. 4B). The results of the RT-qPCR analysis were confirmed by Western blot analysis of KIF18B protein in all cell lines (Fig. 4C, D).

Next, we examined the influence of KIF18B modulation on PCa proliferation and clonal growth using CCK-8 and colony formation assays, respectively. As shown in Fig. 3E, we found that, compared with shCtrl-transduced cells, KIF18B overexpression dramatically and significantly increased the proliferation at day 7 for both DU145 cells (1.58 ± 0.083 vs 2.05 ± 0.090, *P* < 0.001) and PC-3 cells (1.72 ± 0.085 vs 2.24 ± 0.123, *P* < 0.001) (Fig. 4E). In contrast, compared with shCtrl-transduced cells, knockdown of KIF18B with shKIF18B#1 and shKIF18B#2 significantly inhibited the proliferation at day 5 for both DU145 cells (1.48 ± 0.092 vs 0.86 ± 0.062 and 1.02 ± 0.072, respectively; *P* < 0.001) and PC-3 cells (0.623 ± 0.039 vs 0.36 ± 0.051 and 0.50 ± 0.024, respectively; *P* < 0.001) (Fig. 4F). Comparable results were obtained in the colony formation assays. Thus, the number of colonies formed by control and KIF18B-overexpressing cells was 121.7 ± 17.67 and 568.3 ± 28.43, respectively (*P* < 0.001), for DU145 cells and 117 ± 16.82 and 561 ± 25.24, respectively (*P* < 0.001), for PC-3 cells (Fig. 4G). The number of colonies formed by shCtrl-, shKIF18B#1-, and shKIF18B#2-expressing cells was 103.7 ± 27.43 vs 24 ± 12.12 and 55.33 ± 15.14, respectively (*P* < 0.001), for DU145 cells and 117 ± 33.15 vs 28 ± 16.52 and 29 ± 10.82, respectively (*P* < 0.001) for PC-3 cells (Fig. 4H).

### KIF18B promotes the migration and invasion of DU145 and PC-3 cells

**Fig. 5:**
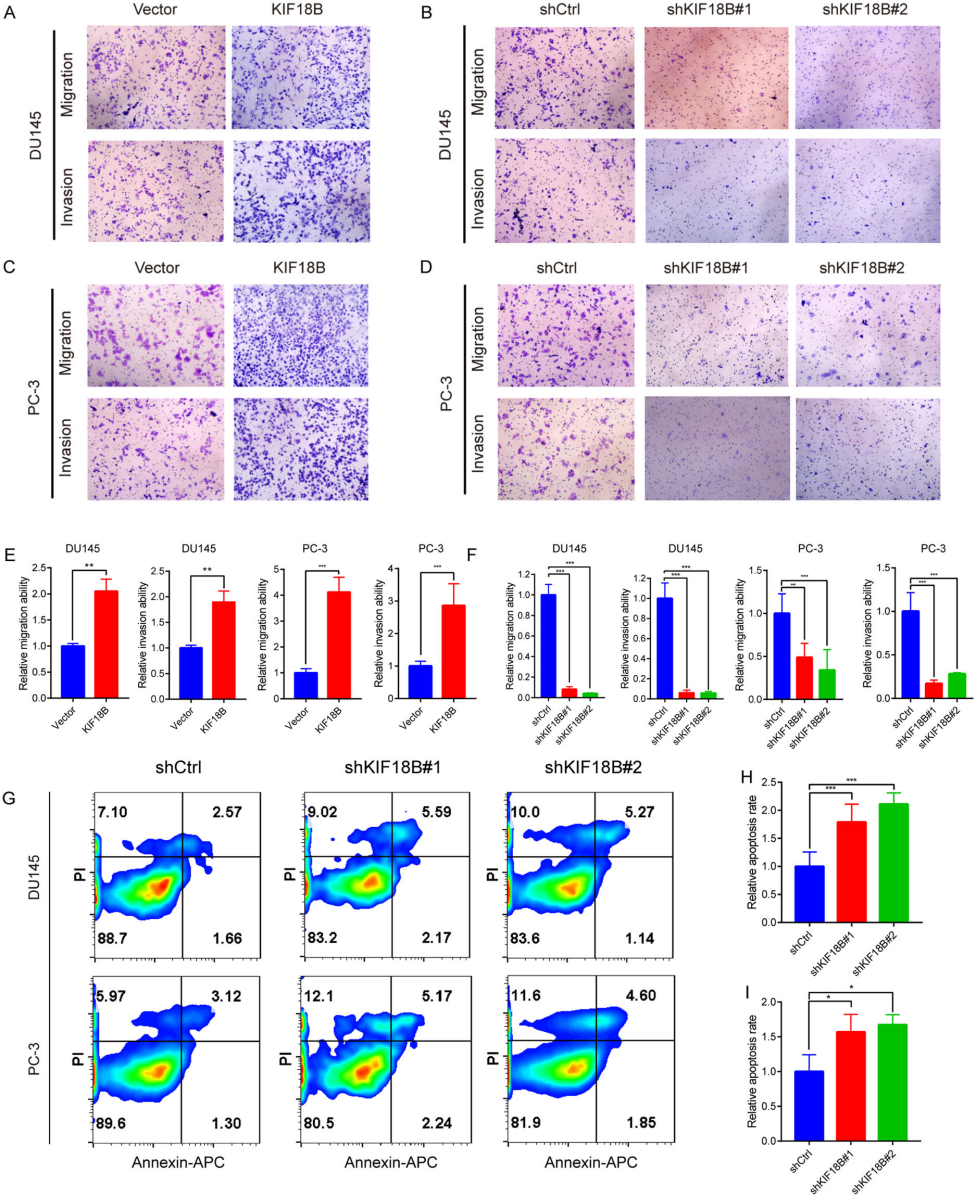
KIF18B promotes migration and invasion and inhibits apoptosis of DU145 and PC-3 cells. **A**–**D** Transwell cell migration and invasion assays of KIF18B-overexpressing DU145 and PC-3 cells, and DU145 and PC-3 cells transfected with shCtrl, shKIF18B#1, or shKIF18B#2. **E**–**F** Statistical analysis of transwell cell migration and invasion ability of KIF18B-overexpressing DU145 and PC-3 cells, and DU145 and PC-3 cells transfected with shCtrl, shKIF18B#1, or shKIF18B#2. **G** PI and annexin V flow cytometric assay of apoptosis of DU145 and PC-3 cells transfected with shCtrl, shKIF18B#1, or shKIF18B#2. **H**, **I** Apoptotic rates of **H** DU145 cells and **I** PC-3 cells transfected with shCtrl, shKIF18B#1, or shKIF18B#2. Data are presented as the mean ± SD of at least three independent experiments. ^*^P < 0.05, ^**^P < 0.01, ^***^P < 0.001 by the Mann–Whitney U test or Student’s *t* test or one-way ANOVA.

We next evaluated the involvement of KIF18B in the migration and invasion behavior of the PCa cells lines using transwell assays. Consistent with the effects of KIF18B modulation on PCa cell proliferation, we found that, compared with shCtrl-transduced cells, KIF18B overexpression significantly increased both the migration (1.00 ± 0.051 vs 1.68 ± 0.068, *P* < 0.01, for DU145 cells and 1.00 ± 0.16 vs 4.12 ± 0.58, *P* < 0.001, for PC-3 cells) and invasion (1.00 ± 0.034 vs 1.71 ± 0.13, *P* < 0.01, for DU145 cells and 1.00 ± 0.15 vs 2.86 ± 0.67, *P* < 0.001, for PC-3 cells) of DU145 and PC-3 cell lines. In contrast, compared with shCtrl-transduced cells, knockdown of KIF18B with shKIF18B#1 and shKIF18B#2 significantly decreased both the migration (1.0 ± 0.07 vs 0.041 ± 0.0023 and 0.05 ± 0.006, respectively, *P* < 0.001, for DU145 cells and 1.00 ± 0.02 vs 0.49 ± 0.16 and 0.34 ± 0.24, *P* < 0.01, *P* < 0.001, for PC-3 cells) and invasion (1.00 ± 0.07 vs 0.029 ± 0.0048 and 0.034 ± 0.015, respectively, *P* < 0.001, for DU145 cells and 1.00 ± 0.21 vs 0.17 ± 0.041 and 0.28 ± 0.015, respectively, *P* < 0.001, for PC-3 cells) of DU145 and PC-3 cell lines (Fig. 5A–F).

### KIF18B inhibits DU145 and PC-3 cell apoptosis

To confirm that KIF18B acts as a growth promoter in PCa, we evaluated the effects of KIF18B knockdown on cell apoptosis using a PI–annexin V flow cytometric assay (Fig. 5G). Notably, KIF18B knockdown resulted in an increase in apoptosis of both DU145 and PC-3 cell lines confirming that KIF18B plays a positive role in the survival and growth of PCa cells. The relative apoptosis rate in shCtrl-, shKIF18B#1-, and shKIF18B#2-expressing cells was 1.00 ± 0.26 vs 1.79 ± 0.32 and 2.11 ± 0.20, respectively (*P* < 0.001), for DU145 cells and 1.00 ± 0.24 vs 1.57 ± 0.25 and 1.67 ± 0.15, respectively (*P* < 0.05) for PC-3 cells (Fig. 5H, I).

### Identification of DEGs associated with KIF18B overexpression in DU145 cells

**Fig. 6:**
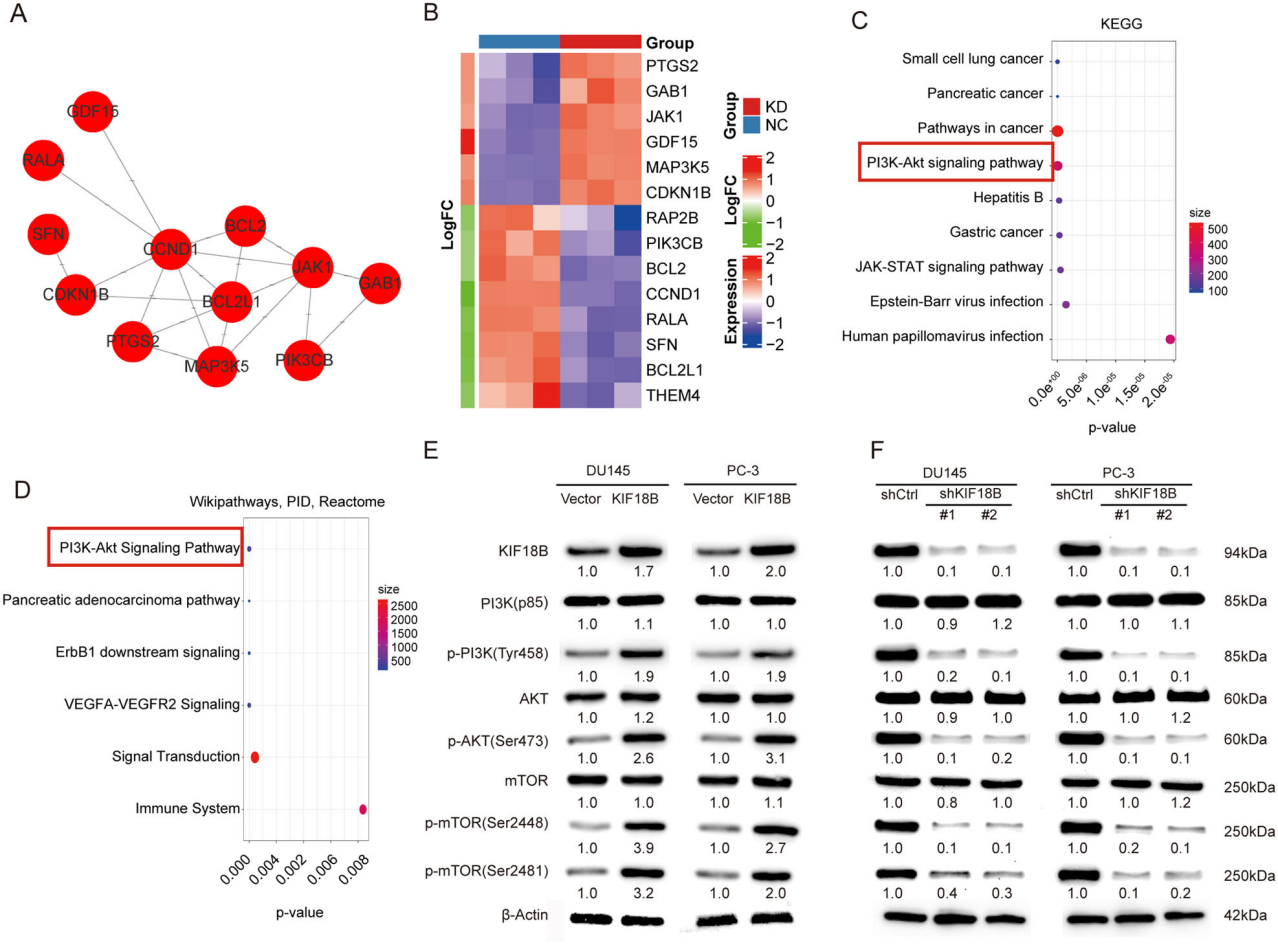
KIF18B activates the PI3K–AKT–mTOR signaling pathway. **A** PPI network of hub genes identified among the DEGs. (**B**) Heatmap of the expression pattern of hub genes. (**C**) and (**D**) Enrichment of DEGs in the PI3K–AKT–mTOR signaling pathway determined using KEGG, WikiPathways, PID, and Reactome databases. (E and F) Western blot analysis of PI3K–AKT–mTOR signaling pathway components in (**E**) KIF18B-overexpressing DU145 and PC-3 cells and (**F**) DU145 and PC-3 cells transfected with shCtrl, shKIF18B#1, or shKIF18B#2. Band intensities of total proteins were normalized to that of β-actin, and band intensities of phosphorylated proteins were normalized to that of the corresponding total proteins.

To explore which genes might be responsible for the effects of KIF18B upregulation in PCa cells, we compared the gene expression profiles of DU145 cells expressing shKIF18B and shCtrl, using a threshold of >1.5-fold (FDR-adjusted *P* < 0.05) as the cutoff for a significantly altered gene. The results of the array experiments identified a total of 1047 DEGs, of which 537 were upregulated and 510 downregulated by KIF18B overexpression. Twelve hub genes were identified from the PPI network constructed from these DEGS (Fig. 6A), and their expression level is shown as a heatmap in Fig. 5B. Pathway enrichment analyses with KEGG, WikiPathways, PID, and Reactome databases revealed significant enrichment of the hub genes in the PI3K–AKT signaling pathway.

### KIF18B activates the PI3K–AKT–mTOR signaling pathway

To validate the in silico findings, we next evaluated how KIF18B overexpression or knockdown influenced the expression of key components of the PI3K–AKT–mTOR signaling pathway by Western blot analysis. The results of this analysis indicated significant upregulation of the activated (phosphorylated) proteins p-PI3K (Tyr458), p-AKT (Ser473), p-mTOR (Ser2448), and p-mTOR (Ser2481) in KIF18B-overexpressing DU145 and PC-3 cells compared with control cells (Fig. 6E). Conversely, expression of the same phosphorylated proteins was significantly downregulated after KIF18B knockdown in both DU145 and PC-3 cells (Fig. 6F).

### KIF18B promotes the growth of tumor xenografts in a mouse model of PCa

**Fig. 7:**
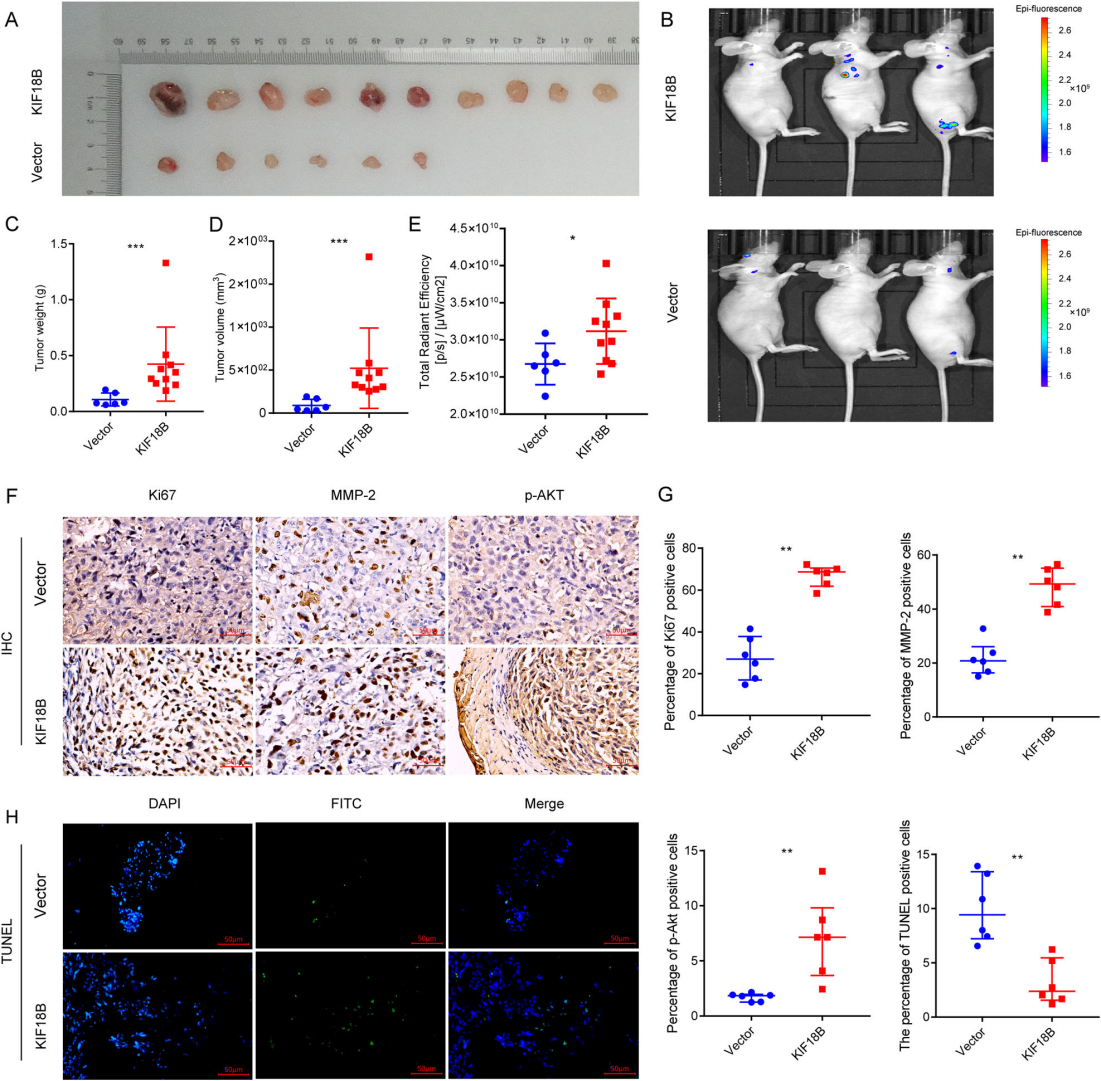
KIF18B promotes PCa tumor growth in vivo. **A** Images of tumors formed from KIF18B-DU145 and Vector-DU145 cells. **B** Representative images of the fluorescence of live animals after injection of Vector-DU145 and KIF18B-DU145 cells. **C**, **D** Average **C** tumor weights (*n* = 16) and **D** tumor volumes (*n* = 16) from animals injected with Vector-DU145 and KIF18B-DU145 cells. **E** Total radiant efficiency of Vector-DU145 and KIF18B-DU145 groups (*n* = 16). **F** Immunohistochemical staining of Ki67, MMP-2, and p-AKT in tumors formed by Vector-DU145 and KIF18B-DU145 cells (*n* = 12). **G** Quantification of protein staining and TUNEL staining. **H** TUNEL assay on tumor tissues from nude mice (*n* = 12). Data are presented as the mean ± SD. Statistical analyses were performed with a *t*-test or *t*-test with Welch’s correction (^*^P < 0.05, ^**^P < 0.01).

The effects of KIF18B on PCa tumor growth in vivo were examined in a mouse xenograft model. Nude mice (*n* = 10/group) were injected subcutaneously with KIF18B-overexpressing or control cells (KIF18B-DU145 and Vector-DU145, respectively), and tumor growth was monitored for 42 days. A total of 10 tumors were obtained from mice injected with KIF18B-DU145 cells and 6 tumors from mice injected with Vector-DU145 cells (no tumors were detected in 4 mice). Analysis of the tumor sizes demonstrated that KIF18B-DU145-derived tumors were larger than those derived from Vector-DU145 cells (Fig. 7A). Representative in vivo images of mice bearing fluorescent tumors on day 42 (Fig. 7B) confirmed that the tumor weight in the KIF18B-DU145-injected group was heavier than those in the Vector-DU145-injected group (median: 0.0775 g for Vector-DU145 *vs* 0.322 g for KIF18B-DU145, *P* < 0.05, Fig. 7C) and the tumor volume was also larger in the KIF18B-DU145-injected group compared with the Vector-DU145-injected group (median: 56.55 mm^3^ for Vector-DU145 *vs* 368.8 mm^3^ for KIF18B-DU145, *P* < 0.001; Fig. 7D). Consistent with these results, the total radiant efficiency was higher for the KIF18B-DU145-injected group than for the Vector-DU145-injected group (mean ± SD: 2.68 × 10^10^ ± 2.78 × 10^9^ f [p/s] / [μW/cm^2^] or Vector-DU145 vs 3.12 × 10^10^ ± 4.42 × 10^9^ [p/s] / [μW/cm^2^] for KIF18B-DU145; *P* < 0.05, Fig. 7E).

### KIF18B promotes tumor cell proliferation and migration, activated AKT, and inhibits apoptosis in PCa xenografts

We next analyzed excised tumor sections for the expression of key proteins involved in AKT pathway signaling, proliferation, migration, and apoptosis using IHC and IF microscopy. Compared with Vector-DU145-derived tumors, KIF18B-DU145 tumors showed an increased percentage of cells expressing Ki67 and MMP-2 (mean ± SD: 27.5 ± 1.5% vs 66.9 ± 5.2% for Ki67, 21.7 ± 6.3% vs 48.4 ± 7.0% for MMP-2; Fig. 7F, G) and an increased percentage of cells expressing p-AKT (median (Q1, Q3): 1.8% (1.3%, 2.0%) for Vector-DU145 vs 7.1% (3.7%, 9.8%) for KIF18B-DU145, Fig. 7F, G). Finally, TUNEL assays on tumor tissues demonstrated a decreased proportion of apoptotic cells in KIF18B-DU145 compared with Vector-DU145 tumors (mean ± SD: 10.0 ± 3.1% for Vector-DU145 vs 3.2 ± 2.1% for KIF18B-DU145, Fig. 7G, H).

## Discussion

Kinesins are ubiquitously expressed in eukaryotic cells, consistent with their vital role in microtubule movement during cell division. KIF18A, a homolog of KIF18B, has been implicated in the formation of medullary chromosomes in mitosis^20^. Abnormalities in sister chromosome separation have been attributed to aberrant expression of KIF18A, leading to cell aneuploidy and, ultimately, tumorigenesis^15^. KIF18A has been implicated in many cancer types, including renal and breast cancer^21,22^.

Expression of KIF18B is upregulated in cervical cancer tissues and cell lines^15^, where it has been shown to promote cell proliferation in vitro and in vivo through the Wnt/β-catenin signaling pathway^15^. To date, however, the expression profile and potential oncogenic role of KIF18B in PCa has remained unclear. In the present study, we provide the first evidence that KIF18B is overexpressed in PCa compared with non-tumor tissues, as initially determined by bioinformatics analysis of a TCGA dataset and then validated by IHC, IF, RT-qPCR, and Western blot analyses of fresh PCa and paracancerous tissues. We also identified an association between high KIF18B expression and poor prostate cancer-free survival based on data from TCGA, and with poor biochemical recurrence-free survival based on clinicopathological data from patients seen at our center. Multivariate Cox proportional hazards analysis demonstrated that KIF18B expression is a significant risk factor for biochemical recurrence-free survival.

Our experiments with human PCa cell lines demonstrated that high KIF18B promotes proliferation, migration, and invasion and inhibits apoptosis of PCa cell lines. These in vitro results were supported by our findings that the growth of PCa xenografts in nude mice was promoted by KIF18B overexpression. Our bioinformatics analyses explored the molecular alterations associated with KIF18B overexpression or knockdown, and revealed some of the downstream molecular processes that couple KIF18B to PCa growth. Of the 1047 DEGs associated with KIF18B overexpression in PCa cells, we identified ten hub genes with high degrees in the PPI network, and pathway analyses revealed enrichment of the genes mainly in the PI3K–AKT signaling pathway. We also found that KIF18B overexpression activates the PI3K–AKT–mTOR signaling pathway in PCa cells in vitro and in tumor xenografts. Collectively, these results suggest that KIF18B is a potential novel biomarker for PCa and offer a theoretical basis for KIF18B-targeted therapy for this disease.

The PI3K–AKT–mTOR pathway is a key oncogenic signaling pathway, not only at the level of tumorigenesis but also in the development of therapy resistance of PCa and additional solid tumors^23–28^. Activation of the PI3K–AKT–mTOR signaling pathway has well-established roles in such critical biological processes as cell proliferation^29^, migration^30^, differentiation^31^, and metabolism^32^. Bitting et al.^33^ demonstrated that inhibitors of PI3K–AKT–mTOR signaling regulation may be novel therapies for castration-resistant PCa. There is evidence for the involvement of kinesin family members in the regulation of various signaling pathways^34^. Pei et al.^34^ showed that KIF11 plays a role in enhancing the self-renewal ability of breast cancer cells via the Wnt/β-catenin pathway, and KIF18B has also been implicated in activation of the Wnt/β-catenin pathway in cervical cancer^15^. However, a role for KIF18B in regulation of this pathway has not previously been demonstrated. In the present study, we provide evidence that expression of p-PI3K (Tyr458), p-AKT (Ser473), p-mTOR (Ser2448), and p-mTOR (Ser2481) is positively regulated by KIF18 expression in DU145 and PC-3 PCa cells, thereby establishing that the role of KIF18B in promoting PCa most likely occurs via PI3K–AKT–mTOR signaling.

There are some limitations to this study. First, only PC-3 and DU145 cell lines were used in this study; however, these cells do not express androgen receptor and are not representative of the majority of primary PCa and castration-resistant PCa. Therefore, our results may not extrapolate to the more common forms of PCa. Second, although we clearly demonstrate that the PI3K–AKT–mTOR pathway is activated in response to stable KIF18B overexpression in PCa cells, we did not identify the underlying mechanism by which this may occur or how it links to the increase in proliferation and invasion observed in KIF18B-overexpressing cells. Further studies will be needed to elucidate these mechanisms.

## Conclusion

This study describes, for the first time, a role for KIF18B in human PCa. KIF18B was overexpressed in clinical specimens, and our in vitro and in vivo analyses demonstrated a positive association between KIF18B expression and PCa cell proliferation, migration, and invasion. Finally, we showed that the growth-promoting effects of KIF18B in PCa are most likely mediated via activation of the PI3K–AKT–mTOR signaling pathway.

## Materials and methods

### Genome expression omnibus dataset

RNA sequencing and clinicopathological data from 113 patients with PCa samples and 13 patients with castration-resistant PCa were downloaded from the Genome Expression Omnibus (GEO; GSE70768). The clinicopathological data included Gleason scores, percentage of tumor burden, age, and prostate-specific antigen (PSA) values.

### Weighted gene co-expression network analysis

Weighted gene co-expression network analysis (WGCNA) was performed as previously described^35^. Briefly, the optimal soft threshold was set at 4 and modules with high similarity of feature genes were combined by a dynamic pruning method. The relationship between clinicopathological data and modules was examined using Pearson’s correlation coefficient. The module with the highest coefficient was selected as the target module. The protein–protein interaction (PPI) network was constructed using genes in the target module according to the rank of degree value calculated by cytohubba in Cytoscape software. The top 30 genes were regarded as the key genes.

### The Cancer Genome Atlas dataset

A PCa dataset from The Cancer Genome Atlas (TCGA) was analyzed using the UALCAN^36^ online tool, which enables analysis of survival and identification of novel gene expression patterns in tumor subgroups. Level 3 RNA-seq and clinical data from PCa patients were used in the analyses.

### Validation and correlation analysis of key genes

Validation of the identified key genes in the TCGA dataset, co-expression analysis, and expression correlation analysis of these genes were performed using the online tool GEPIA2 (http://gepia2.cancer-pku.cn/).

### Institutional patients and samples

This study was approved by the Ethics Committee of the First Affiliated Hospital of Fujian Medical University, and informed consent was obtained from all patients before sample collection. Fresh paracancerous and PCa tissue samples for RT-qPCR and Western blot analyses were obtained from 18 PCa patients treated at the Department of Urology, First Affiliated Hospital of Fujian Medical University. Paracancerous tissues were obtained 2 cm from the edge of the PCa tissues. Clinicopathological data and biochemical recurrence time were collected.

### Immunohistochemistry and immunofluorescence microscopy

Immunohistochemistry (IHC) and immunofluorescence (IF) microscopy were performed using standard procedures. The primary anti-KIF18B antibody was used at a 1:10 dilution and was obtained from Abcam (Cambridge, UK). Protein expression in tissues was evaluated according to the percentage of positively stained cells (≤0, 5%; 1, 6–25%; 2, 26–50%; 3, 51–75%; and 4, ≥76%) and the staining intensity (0, no color; 1, mild; 2, moderate; and 3, strong).

### Cell lines and reagents

DU145, PC-3, and LNCaP cell lines were obtained from the American Type Culture Collection (ATCC). Cells were cultured in Dulbecco’s modified Eagle’s medium (DMEM) supplemented with 10% fetal bovine serum (FBS; A11-102, Ausbian, Australia) and 1% streptomycin and penicillin at 37 °C in a 5% CO_2_ humidified atmosphere. Anti-PI3K (p85), anti-p-PI3K (Tyr458), anti-AKT, anti-p-AKT (Ser473), anti-mTOR, anti-p-mTOR (Ser2448), anti-Ki67, anti-MMP-2, anti-p-mTOR (Ser2481) were purchased from Cell Signaling Technology (Danvers, MA), and anti-KIF18B was purchased from Abcam (Cambridge, UK).

### Vector construction and lentiviral infection

The coding sequence of KIF18B (National Center for Biotechnology Information) was synthesized and inserted into pcDNA 3.1 vector (Invitrogen; Thermo Fisher Scientific, Inc.). Lentivirus packaging was performed by GeneChem Co. Ltd. (Shanghai, China). PCa cells were infected by lentivirus particles following the manufacturer’s instruction. DU145 and PC-3 cells stably transduced with the KIF18B expression vector (KIF18B-DU145, KIF18B-PC-3) or control vector (Vector-DU145 and Vector-PC-3) were obtained by selection with 1 µg/ml puromycin.

### RNA interference

Two short hairpin RNAs (shRNAs) targeting KIF18B and a control shRNA were synthesized by GeneChem (Shanghai, China). Control sequence: 5′-TTCTCCGAACGTGTCACGT-3′; shKIF18B#1: 5′-GCGCTCATCAACGTCCTCAAT-3′; and shKIF18B#2: 5′-AGGCTCTCGCTGAAGAGCAAT′-3′.

### Reverse-transcription quantitative PCR (RT-qPCR)

Total RNA was extracted and reverse transcribed (M1705, Promega). qPCR was performed using SYBR Premix Ex Taq II kit (DRR041B; TAKARA, Shanghai, China) and a LightCycler 480 thermocycler (Roche, Basel, Switzerland) with the following conditions: 94 °C for 3 min, 22 cycles of 94 °C for 30 s, 55 °C for 30 s, and 72 °C for 30 s, followed by 72 °C for 5 min. The ΔΔCq method was used to analyze the data, and glyceraldehyde 3-phosphate dehydrogenase (GAPDH) was amplified as an internal control^37^. The qPCR primers for *KIF18B* were: forward 5′-CCTATTTCCCATGATTCCTTCATA-3′ and reverse 5′-GTAATACGGTTATCCACGCG-3′.

### Western blot analysis

Cells were lysed in RIPA buffer (P0010S; Beyotime, Shanghai, China), and proteins were quantified with a BCA assay kit (P0013B, Beyotime). Proteins (20 μg per lane) were resolved by 10% SDS-PAGE and transferred to PVDF membranes. Membranes were blocked by incubation in Tris-buffered saline containing 0.1% Tween-20 (TBST) and 5% milk for 1 h at room temperature, washed, and then incubated for 24 h at 4 °C with rabbit anti-KIF18B (1:5000 dilution; ab168812, Abcam) or mouse anti-GAPDH (1:2000; sc-32233, Santa Cruz Biotechnology, Shanghai, China). The membranes were washed three times with TBST, incubated for 1 h at room temperature with HRP-conjugated goat anti-mouse IgG (1:2000; SA00001-1, Proteintech Group, Inc., Chicago, IL, USA), and developed using ECL reagent (M3121/1859022; Thermo Fisher Scientific).

### Colony formation assays

Single-cell suspensions of DU145 and PC-3 were seeded into 6-well plates at 10^3^ cells/well and incubated at 37 °C for at least 14 days. The cells were fixed with 4% formaldehyde for 30 min, incubated with 0.1% crystal violet solution for 30 min, rinsed with tap water, air dried, and photographed. The number of stained colonies in each well was counted.

### CCK-8 viability assays

Viability was measured using a CCK-8 assay kit (Abmole Bioscience, Shanghai, China). DU145 and PC-3 cells were seeded into 96-well plates at 10^3^ cells/well and incubated for the indicated times. At the end of the culture period, 10 µl of CCK-8 solution was added to each well and the plates were incubated at 37 °C for 1 h. The absorbance at 490 nm was measured using a microplate reader (M2009PR, Tecan infinite).

### Transwell migration and invasion assays

DU145 and PC-3 cells were resuspended in growth medium containing 10% FBS and placed in the upper chamber inserts of transwell plates (24-well, 8.0-μm pore diameter; #3422, Corning, Merck Life Science, Shanghai, China) at 1 × 10^5^ cells/600 μl/well for both migration and invasion assays, except that the inserts were precoated with 20 μg Matrigel (BD Biosciences, Oxford, UK) in culture medium for the invasion assay. The same volume of culture medium with 30% FBS was added to the lower chamber inserts and the plates were incubated for 6 h at 37 °C. The culture medium was discarded, and cells remaining attached to the membrane were incubated with Giemsa stain (#32884, Sigma) for 3 min at room temperature and then imaged at 200× magnification using a light microscope (Caikon, Shanghai Caikon Optical Instrument Co., Ltd., Shanghai, China).

### Apoptosis assay

Apoptosis assays were performed by staining the cells with propidium iodide (PI) and annexin V-APC (BD Biosciences) followed by analysis by flow cytometry.

### Gene expression microarray analysis

Total RNA was extracted with TRIzol reagent (Shanghai GeneChem). RNA quality was analyzed utilized a Nanodrop 2000 spectrophotometer (1.7 < A260/A280 < 2.2) and an Agilent 2100 Bioanalyzer and Agilent RNA 6000 Nano Kit (RNA ≥ 7.0, 28 S/18 S > 0.7; Agilent, Santa Clara, CA, USA). Reverse transcription was performed using a GeneChip 3′ IVT PLUS Kit (Thermo Fisher Scientific) and array hybridization, washing, and staining were performed with a GeneChip Hybridization Wash and Stain Kit. Affymetrix GeneChip^®^ PrimeView™ Human Gene Expression Arrays (Thermo Fisher Scientific) were used for screening. A log2 scale was used to normalize raw data obtained from the GeneChip Scanner 3000 system. We used linear models and empirical Bayes methods to calculate *P* values, and false discovery rate (FDR)-adjusted *P* values were determined using the Benjamini–Hochberg method. Genes with a fold change in expression of >1.5 with an FDR-adjusted *P* value of <0.05 were considered significantly differentially expressed genes (DEGs).

### Selection of hub genes

The online search tool STRING was used to determine the interaction of DEGs and to construct a PPI network of hub genes. Heatmaps were used to demonstrate the expression patterns of hub genes. Gene enrichment analyses were performed using KEGG, WikiPathways, PID, and Reactome databases.

### In vivo experiments

Nude mice were randomly divided into two groups (*n* = 10 per group). Four-week-old male nude mice (BALB/c) were injected with DU145 cells (2 × 10^6^ cells in 200 μl) subcutaneously into the right forelimb armpit, and tumor volumes were monitored for a maximum of 42 days. The following formula was used: tumor volume (mm^3^) = width (mm^2^) × length (mm) × 0.5. The fluorescent signal of GFP were detected. The mice were anesthetized with 2% isoflurane and analyzed using an IVIS Lumina II In Vivo Imaging System (Perkin Elmer) with Live Imaging Acquisition and Analysis software. The mice were then sacrificed, and tumors were excised for analysis.

### Terminal deoxynucleotidyl transferase dUTP nick-end labeling (TUNEL) assay

Analysis of apoptotic cells in paraffin sections of excised tumors was performed using a fluorometric TUNEL kit (KeyGEN BioTECH, Jiangsu, China, KGA7071) according to the manufacturer’s protocol.

### Statistical analysis

Data were analyzed using Student’s *t* test, Chi-square test, one-way ANOVA, Bonferroni’s post hoc test, Mann–Whitney U test, and log-rank test as indicated in the figure legends. All analyses were performed with SPSS 19.0 software (IBM, Armonk, NY, USA) and Prism version 6.0 (GraphPad Software, San Diego, CA, USA). A *P* value <0.05 was considered statistically significant. For the bar and line graphs, data are presented as the mean ± standard deviation (SD) from at least three independent experiments. For immunoblot quantification, the gels were scanned, and the band intensities were examined with ImageJ software. For quantification of total protein, the band intensity was normalized to that of the loading control, and for quantification of phosphorylated protein, the band intensity was normalized to the corresponding total protein intensity.

## Supplementary information

Supplementary Figure 1

Supplementary Figure 2

## References

[1] Siegel RL, Miller KD, Jemal A (2020). Cancer statistics, 2020. CA.

[2] Das R (2017). MicroRNA-194 promotes prostate cancer metastasis by inhibiting SOCS2. Cancer Res..

[3] Smith RA (2019). Cancer screening in the United States, 2019: a review of current American Cancer Society guidelines and current issues in cancer screening. CA.

[4] Yang Y (2020). Kinesin family member 3A inhibits the carcinogenesis of non-small cell lung cancer and prolongs survival. Oncol. Lett..

[5] Li D, Sun H, Meng L, Li D (2020). The overexpression of kinesin superfamily protein 2A (KIF2A) was associated with the proliferation and prognosis of esophageal squamous cell carcinoma. Cancer Manag. Res..

[6] Liu G (2020). The kinesin motor protein KIF4A as a potential therapeutic target in renal cell carcinoma. Investig. N. Drugs.

[7] De S, Cipriano R, Jackson MW, Stark GR (2009). Overexpression of kinesins mediates docetaxel resistance in breast cancer cells. Cancer Res..

[8] Ishikawa K (2008). Mitotic centromere-associated kinesin is a novel marker for prognosis and lymph node metastasis in colorectal cancer. Br. J. Cancer.

[9] Hirokawa N, Takemura R (2004). Kinesin superfamily proteins and their various functions and dynamics. Exp. Cell Res..

[10] McHugh T, Gluszek AA, Welburn JPI (2018). Microtubule end tethering of a processive kinesin-8 motor Kif18b is required for spindle positioning. J. Cell Biol..

[11] Rath O, Kozielski F (2012). Kinesins and cancer. Nat. Rev. Cancer.

[12] Yao, D. W., Song, Q. & He, X. Z. Kinesin family member 23 (KIF23) contributes to the progression of bladder cancer cells in vitro and in vivo. *Neoplasma* (2020). 10.4149/neo_2020_200803N808. 10.4149/neo_2020_200803N808 33231086

[13] Wang ZX, Ren SC, Chang ZS, Ren J (2020). Identification of kinesin family member 2A (KIF2A) as a promising therapeutic target for osteosarcoma. BioMed. Res. Int..

[14] Itzel T (2015). Translating bioinformatics in oncology: guilt-by-profiling analysis and identification of KIF18B and CDCA3 as novel driver genes in carcinogenesis. Bioinformatics.

[15] Wu Y (2018). KIF18B promotes tumor progression through activating the Wnt/beta-catenin pathway in cervical cancer. OncoTargets Ther..

[16] Fan HT, Shi YY, Lin Y, Yang XP (2019). EHMT2 promotes the development of prostate cancer by inhibiting PI3K/AKT/mTOR pathway. Eur. Rev. Med. Pharmacol. Sci..

[17] Torrealba, N., et al. TGF-β/PI3K/AKT/mTOR/NF-kB pathway. Clinicopathological features in prostate cancer. *Aging Male* 1–11 (2019). 10.1080/13685538.2019.1597840.

[18] Lamoureux F, Zoubeidi A (2013). Dual inhibition of autophagy and the AKT pathway in prostate cancer. Autophagy.

[19] Kreisberg JI (2004). Phosphorylation of Akt (Ser473) is an excellent predictor of poor clinical outcome in prostate cancer. Cancer Res..

[20] Gardner MK, Odde DJ, Bloom K (2008). Kinesin-8 molecular motors: putting the brakes on chromosome oscillations. Trends Cell Biol..

[21] Chen QI (2016). Elevated expression of KIF18A enhances cell proliferation and predicts poor survival in human clear cell renal carcinoma. Exp. Ther. Med..

[22] Kasahara M (2016). Clinicopathological relevance of kinesin family member 18A expression in invasive breast cancer. Oncol. Lett..

[23] Aoki M, Fujishita T (2017). Oncogenic roles of the PI3K/AKT/mTOR Axis. Curr. Top. Microbiol. Immunol..

[24] Marquard FE, Jücker M (2020). PI3K/AKT/mTOR signaling as a molecular target in head and neck cancer. Biochem. Pharmacol..

[25] Braglia, L., Zavatti, M., Vinceti, M., Martelli, A. M. & Marmiroli, S. Deregulated PTEN/PI3K/AKT/mTOR signaling in prostate cancer: still a potential druggable target? *Biochim. et Biophys. Acta Mol. Cell Res.* 2020:118731. 10.1016/j.bbamcr.2020.118731. 10.1016/j.bbamcr.2020.118731 32360668

[26] Kolinsky MP (2020). A phase I dose-escalation study of enzalutamide in combination with the AKT inhibitor AZD5363 (capivasertib) in patients with metastatic castration-resistant prostate cancer. Ann. Oncol..

[27] Huang JB (2020). Up-regulation of LIMK1 expression in prostate cancer is correlated with poor pathological features, lymph node metastases and biochemical recurrence. J. Cell Mol. Med..

[28] Fu, R. & Tong, J. S. miR-126 reduces trastuzumab resistance by targeting PIK3R2 and regulating AKT/mTOR pathway in breast cancer cells. *J. Cell. Mol. Med.* **24**, 7600–7608 (2020). 10.1111/jcmm.15396 PMC7339158 32410348

[29] Zhang, L., Ge, S. & Cao, B. Long non-coding RNA MIAT promotes cervical cancer proliferation and migration. *J. Biochem.* **168**, 183–190 (2020). 10.1093/jb/mvaa037 32239132

[30] Wu, Y. J., Lin, S. H., Din, Z. H., Su, J. H. & Liu, C. I. Sinulariolide inhibits gastric cancer cell migration and invasion through downregulation of the EMT process and suppression of FAK/PI3K/AKT/mTOR and MAPKs signaling pathways. *Mar. Drugs* **17**, 668 (2019). 10.3390/md17120668 PMC6950622 31783709

[31] Wu XL (2018). Effects of Glut1 gene silencing on proliferation, differentiation, and apoptosis of colorectal cancer cells by targeting the TGF-β/PI3K-AKT-mTOR signaling pathway. J. Cell. Biochem..

[32] Ramapriyan R (2019). Altered cancer metabolism in mechanisms of immunotherapy resistance. Pharmacol. Ther..

[33] Bitting RL, Armstrong AJ (2013). Targeting the PI3K/Akt/mTOR pathway in castration-resistant prostate cancer. Endocr. Relat. Cancer.

[34] Pei YY (2019). Kinesin family member 11 enhances the self-renewal ability of breast cancer cells by participating in the Wnt/β-catenin pathway. J. Breast Cancer.

[35] Wu YP (2020). Identification of key genes and pathways in castrate-resistant prostate cancer by integrated bioinformatics analysis. Pathol. Res. Pract..

[36] Chandrashekar DS (2017). UALCAN: a portal for facilitating tumor subgroup gene expression and survival analyses. Neoplasia.

[37] Livak KJ, Schmittgen TD (2001). Analysis of relative gene expression data using real-time quantitative PCR and the 2(-Delta Delta C(T)) method. Methods.

