# Supplementary figures and images for: Kinesin family member 18B regulates the proliferation and invasion of human prostate cancer cells
Source: Cell Death Dis. 2021 Mar 22;12(4):302. doi: 10.1038/s41419-021-03582-2 (PMC7985494; doi:10.1038/s41419-021-03582-2)

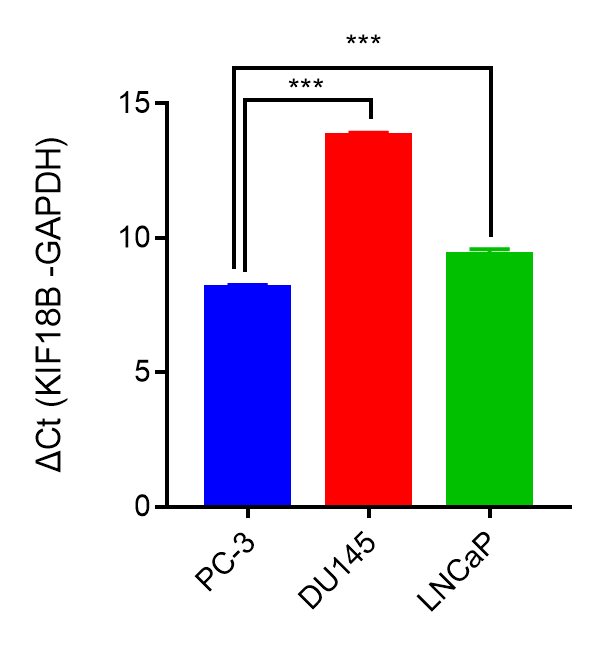

Supplement: Supplementary file 1 — Supplementary Figure 1 [file 41419_2021_3582_MOESM1_ESM.tif]

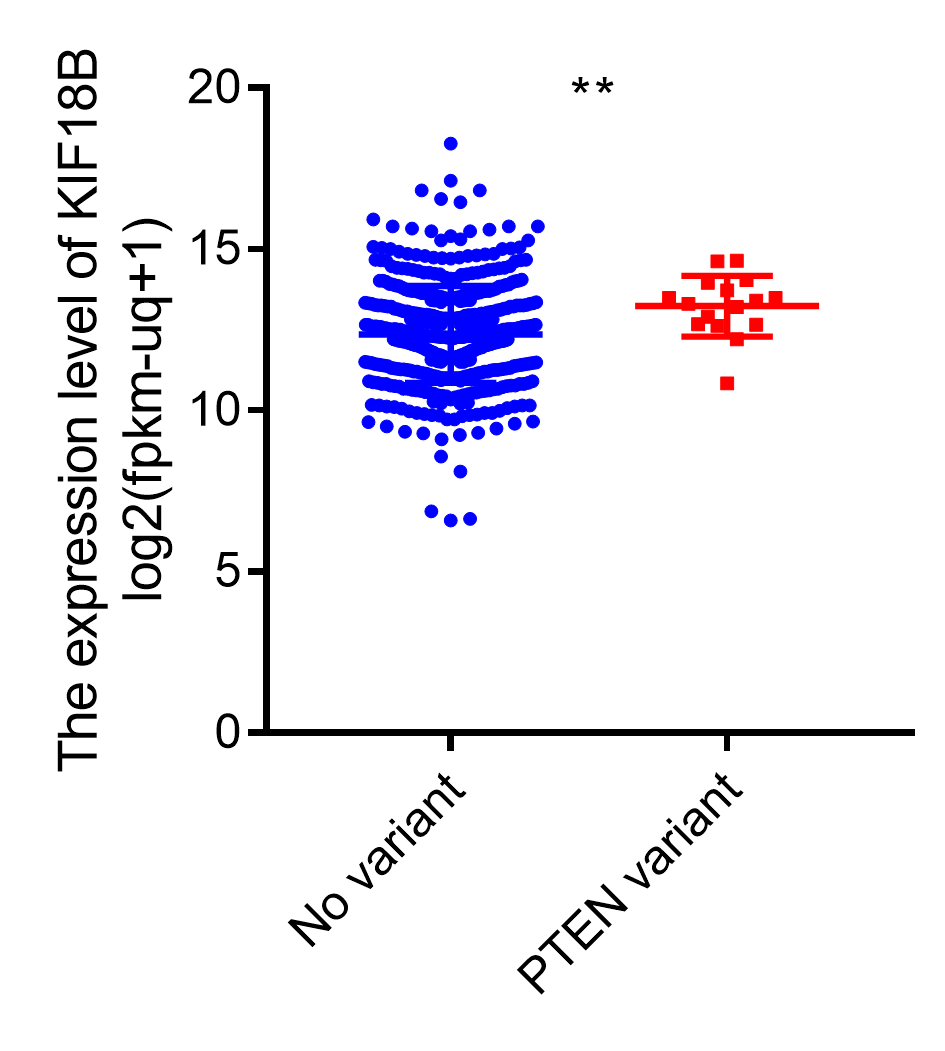

Supplement: Supplementary file 2 — Supplementary Figure 2 [file 41419_2021_3582_MOESM2_ESM.tif]
